# Survival Outcomes After irAE-Related Nivolumab Discontinuation in mNSCLC: A Multicenter Study

**DOI:** 10.3390/jcm15082818

**Published:** 2026-04-08

**Authors:** Aysegul Merc Cetinkaya, Mehmet Haluk Yucel, Latif Karahan, Mustafa Ersoy, Ahmet Bilici, Mustafa Erman, Ali Murat Tatli, Sema Sezgin Goksu

**Affiliations:** 1 Department of Medical Oncology, Akdeniz University Faculty of Medicine Hospital, Antalya 07070, Turkey; semasezgingoksu@gmail.com; 2 Department of Medical Oncology, Medipol University Faculty of Medicine Hospital, Istanbul 34214, Turkey; mhalukyucel@gmail.com (M.H.Y.);; 3 Department of Medical Oncology, Hacettepe University Institute of Oncology, Ankara 06230, Turkey; lkarahanmd@gmail.com (L.K.);; 4 Department of Medical Oncology, Kutahya Health Sciences University Faculty of Medicine Hospital, Kutahya 43100, Turkey; mustafa.ersoy@ksbu.edu.tr; 5 Department of Medical Oncology, Memorial Hospital, Antalya 07025, Turkey; alimurattat@hotmail.com

**Keywords:** non-small cell lung cancer, nivolumab, immune-related adverse events, progression-free survival, overall survival, real-world data

## Abstract

**Background**: Immune checkpoint inhibitors (ICIs) such as nivolumab have transformed the treatment landscape of metastatic non-small cell lung cancer (NSCLC). We aimed to evaluate survival outcomes of patients who developed immune-related adverse events (irAEs) that required permanent treatment discontinuation. **Methods**: This national, multicenter study included 25 patients with metastatic NSCLC from four tertiary oncology centers in Turkey. All patients received nivolumab monotherapy and discontinued treatment because of irAEs. Long-term survival was analyzed using Kaplan–Meier methods and compared indirectly with historical benchmarks. **Results**: The median overall survival (OS) was 35.73 months (95% CI: 30.06–41.40). The 2-year and 5-year OS rates were 78.9% and 27.0%, respectively. Median progression-free survival (PFS) was 16.23 months. Pneumonitis was the most frequent irAE (48%). Liver metastases significantly reduced OS (23.93 vs. 38.50 months, *p* = 0.005). In univariate analysis, ECOG 2 status (HR:22.07), bone metastases (HR:3.52), and subsequent systemic therapy (HR:30.19) weresignificant predictors of progression. **Conclusions**: Patients with metastatic NSCLC who discontinue nivolumab due to irAEs achieve notable survival outcomes, suggesting that treatment-limiting toxicity may signal a robust and durable antitumor immune response. These findings, though limited by a small cohort, highlight a distinct responder phenotype in real-world clinical practice.

## 1. Introduction

The advent of immune checkpoint inhibitors (ICIs), particularly programmed cell death-1 (PD-1) inhibitors such as nivolumab, has fundamentally transformed the treatment landscape of metastatic non-small cell lung cancer (NSCLC). Despite the durable survival benefit demonstrated in pivotal clinical trials, including CheckMate 017 and CheckMate 057, a substantial proportion of patients fail to achieve long-term benefits, highlighting the unmet need for reliable and easily applicable clinical biomarkers [1,2,3]. The development of immune-related adverse events (irAEs) is often considered an off-target effect of an activated immune system, suggesting a potential correlation between toxicity and treatment efficacy [4,5,6]. This association is hypothesized to stem from shared antigens between the tumor and healthy tissues, where an overactive immune response targets both, thereby signaling a more robust anti-tumor effect [5].

Currently, programmed death-ligand 1 (PD-L1) expression and tumor mutational burden (TMB) are the most widely used predictive biomarkers for immunotherapy; however, their predictive performance remains suboptimal and inconsistent in real-world clinical practice. In this context, increasing attention has been directed toward immune-related adverse events (irAEs), which are thought to reflect heightened immune activation and may serve as surrogate markers of antitumor immune response [6,7,8]. The systematic review by Petrelli et al. has further solidified this link, showing that the presence of irAEs is significantly associated with improved progression-free and overall survival across various cancer types [4].

While long-term follow-up of pivotal trials suggests a positive association between irAEs and survival [3,9], real-world data—especially for patients forced to permanently discontinue therapy—remains limited [10,11]. Managing the delicate balance between therapeutic benefit and severe toxicity remains a major challenge in clinical oncology. Patients who must stop treatment early due to irAEs represent a unique subgroup where the “primed” immune system may continue to exert anti-tumor pressure even in the absence of continued drug exposure [7,12]. This study evaluated survival outcomes in a multicenter, real-world setting for patients with metastatic NSCLC who developed irAEs during nivolumab monotherapy and discontinued treatment as a result.

## 2. Materials and Methods

This national, multicenter, retrospective study included patients from four tertiary oncology centers in Turkey treated between 2015–2024. The eligibility criteria included patients aged ≥18 years with histologically confirmed stage 4 NSCLC. To be included in the study, patients must have received at least one dose of nivolumab monotherapy, experienced permanent treatment discontinuation specifically due to irAE(s), and had complete follow-up data available in medical records. Patients receiving combination immunotherapy or those with insufficient follow-up data were excluded. The study was designed as a descriptive multicenter experience to evaluate long-term outcomes in this specific patient subgroup.

Clinicopathological data were collected from electronic medical records. The collected data included age, gender, smoking history, ECOG performance status, histological subtype, presence of brain or liver metastases, and the line of therapy. IrAEs were defined as suspected immunologic etiologies requiring monitoring or immunosuppressive treatment and were graded according to CTCAE v5.0. While all types of immune-related toxicities leading to permanent discontinuation were documented, clinically significant events such as pneumonitis, colitis, and hepatitis were closely monitored due to their potential for severe morbidity in this patient population.

The primary endpoint was progression-free survival (PFS), and the secondary endpoint was overall survival (OS). PFS was defined as the time from the first dose of nivolumab to disease progression or death from any cause. OS was defined as the time from the first dose of nivolumab to death from any cause.Survival curves were estimated using the Kaplan–Meier method with log-rank tests. Due to the small sample size (*n* = 25), statistical analysis was primarily limited to univariate methods to avoid model instability and overfitting. Variables with a *p*-value < 0.1 in univariate screening were further evaluated to identify clinical trends. A two-sided *p*-value < 0.05 was considered significant.

During the preparation of this manuscript, the authors utilized Artificial Intelligence (AI) specifically for language editing, stylistic refinement, translation of data tables and assistance with reference formatting. These tools were employed solely to enhance the clarity, readability, and academic flow of the text. All scientific content, data interpretation, and conclusions were developed and verified by the authors. The authors take full responsibility for the accuracy and integrity of the manuscript.

This study was conducted in accordance with the principles of the Declaration of Helsinki and Good Clinical Practice guidelines. Approval was obtained from the Akdeniz University Faculty of Medicine Clinical Research Ethics Committee (Date: 31 October 2024; Decision No: 722). Owing to the retrospective nature of the study, the requirement for individual informed consent was waived by the ethics committee. To support the reproducibility of our findings, de-identified data will be made available to other researchers upon reasonable request.

## 3. Results

A total of 25 patients were included. The median age was 62 years (range: 39–81), with 60% being over age 60. Most patients had an Eastern Cooperative Oncology Group (ECOG) Performance Status (PS) score of 0–1 (92%). Adenocarcinoma was the most common histological type (56%). Metastatic sites included lung (68%), bone (48%), liver (24%), and CNS (24%). De novo metastatic disease was present in 64% of cases. Regarding PD-L1 levels, 45.8% were <1%, 16.7% were 1–49%, and 16.7% were >49% (Table 1).

The most frequent irAE was pneumonitis (48%), followed by colitis (24%) and dermatologic toxicity (12%). Other events included adrenalitis, hepatitis, myositis, and hematologic toxicity (4% each). The severity was grade 2 in 44%, grade 3 in 44%, and grade 4 in 8% of the irAEs (Table 1).

**Figure 1:**
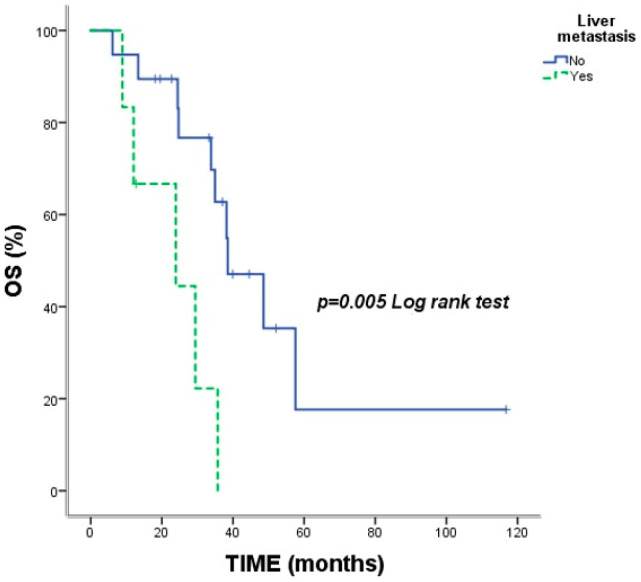
Kaplan–Meier survival curves according to liver metastasis status. Vertical tick marks indicate censored observations. *p*-value was calculated using the log-rank test.

**Figure 2:**
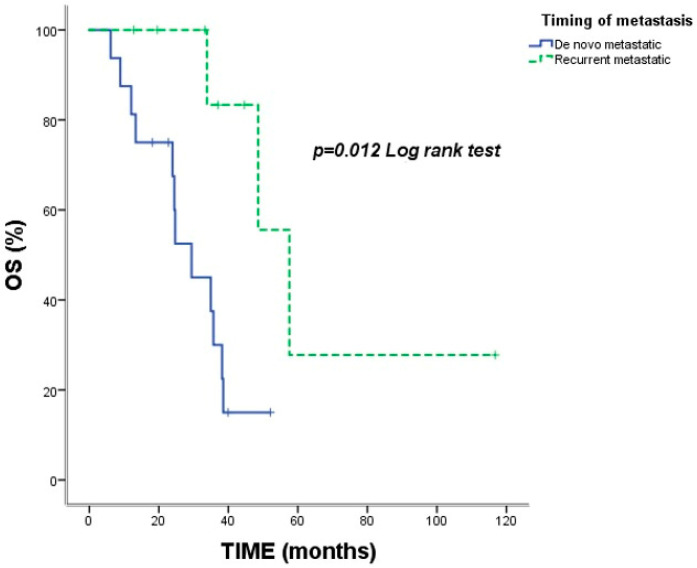
Kaplan–Meier overall survival (OS) curves according to timing of metastasis (de novo vs. recurrent). Vertical tick marks indicate censored observations. The *p*-value was calculated using the log-rank test.

Median OS for the entire cohort was 35.73 months (95% CI: 30.06–41.40). The estimated 2-year and 5-year OS rates were 78.9% and 27.0%, respectively. Significantly shorter OS was observed in patients with liver metastases (23.93 vs. 38.50 months; *p* = 0.005) (Figure 1) and de novo metastatic disease (29.43 vs. 57.56 months; *p* = 0.012) (Figure 2) (Table 2).

**Figure 3:**
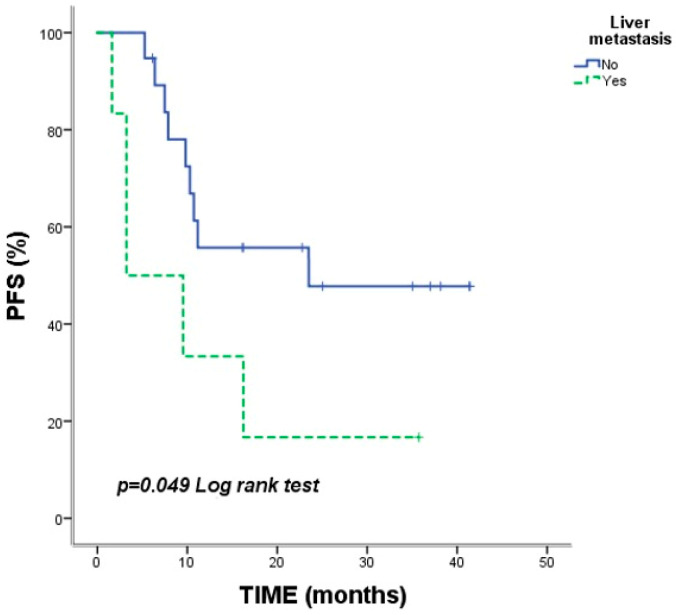
Kaplan–Meier progression-free survival (PFS) curves according to liver metastasis status. Vertical tick marks indicate censored observations. The *p*-value was calculated using the log-rank test.

**Figure 4:**
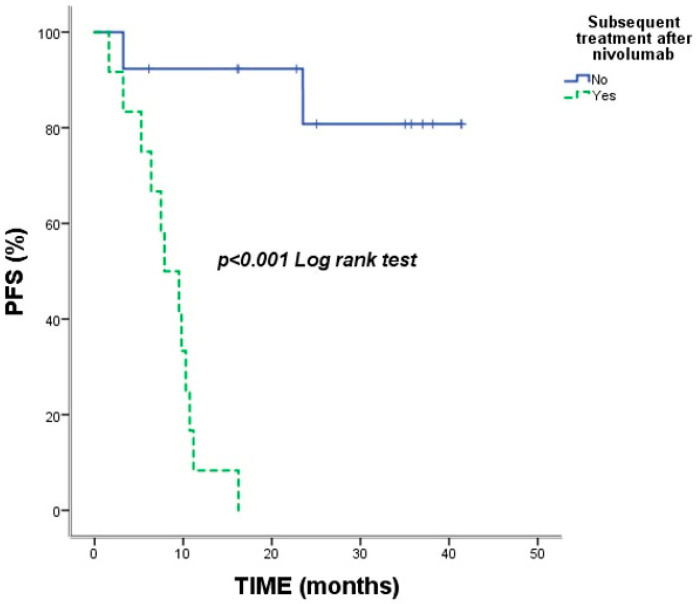
Kaplan–Meier curves for progression-free survival (PFS) according to subsequent treatment after nivolumab (yes vs. no). Vertical tick marks represent censored observations (patients without progression or lost to follow-up at the time of analysis). The *p*-value was derived from the log-rank test.

Median PFS was 16.23 months (95% CI: 2.05–30.40), with a 2-year PFS rate of 40.1%. Inferior PFS was associated with ECOG PS score of 2 (*p* = 0.001), liver metastases (*p* = 0.049) (Figure 3), subsequent treatment after nivolumab (*p* < 0.001) (Figure 4) and bone metastases (*p* = 0.018). Patients with a partial response to nivolumab had significantly longer PFS (*p* = 0.009) (Table 3).

In the univariate analysis, several clinical factors were associated with a significantly increased risk of disease progression. These factors included an ECOG PS score of 2 (*p* = 0.015), the presence of bone metastases (*p* = 0.026), and the requirement for subsequent systemic therapy following nivolumab discontinuation (*p* = 0.001) (Table 4). Given the small cohort size (*n* = 25), these variables were interpreted as descriptive indicators of clinical outcome rather than independent predictors in a multivariate model, to avoid the risk of statistical overfitting.

## 4. Discussion

In this real-world, multicenter study, patients with metastatic NSCLC who discontinued nivolumab due to irAEs demonstrated a median OS of 35.73 months and a 5-year OS rate of 27.0%. These results are numerically superior to landmark trials, such as CheckMate 017 and 057, where the 5-year OS for nivolumab-treated patients was approximately 13.4% [3]. Our observed 2-year PFS rate of 40.1% and 2-year OS of 78.9% also exceed the outcomes reported in these pivotal trials [1,2,3]. This notable difference suggests that patients who develop treatment-limiting toxicity may represent a distinct “prolonged-responder” phenotype. These findings align with Haratani et al. and recent meta-analyses, which identified irAE development as a clinical surrogate for robust anti-tumor immunity [4,6].

The survival benefit observed despite permanent treatment discontinuation supports the “hit-and-run” hypothesis. It has been suggested that once the immune system is sufficiently primed and achieves a “therapeutic threshold”—as evidenced by clinical irAEs—the induced T-cell memory appears capable of sustaining long-term disease control without the need for continuous PD-1 inhibition [7,10,11,12]. This ‘memory’ effect may explain the long-term survival plateaus observed in our cohort despite the early cessation of therapy [5,7,12].

In our cohort, pneumonitis was the most frequent irAE (48%), and these patients achieved a noteworthy median OS of 48.56 months. This is consistent with findings by Suresh et al., indicating that appropriately managed ICI-induced pneumonitis can correlate with favorable clinical outcomes [13]. Conversely, even within this immune-reactive cohort, liver metastases remained a negative prognostic indicator (mOS 23.9 months), likely due to the liver’s unique immunosuppressive microenvironment [14]. Furthermore, we observed that patients requiring rapid initiation of subsequent systemic therapy following discontinuation had poorer outcomes. This likely reflects a more aggressive underlying tumor biology in a subset of patients who fail to achieve the sustained ‘hit-and-run’ effect typically seen in long-term survivors [15].

Our findings align with several large-scale studies and systematic reviews that have reported a positive association between irAE development and improved survival in NSCLC [4,8,9]. For instance, Santini et al. demonstrated that patients experiencing irAEs had significantly longer OS compared to those who did not [8]. Similarly, Owen et al. highlighted that this association remains consistent across different types of immune checkpoint inhibitors [9]. The comprehensive review by Michot et al. further underscores the complex but often beneficial relationship between immune-mediated toxicity and anti-tumor efficacy [7].

Despite these encouraging observations, our study has several limitations. The retrospective, single-arm design and the relatively small sample size (*n* = 25) limit the generalizability of the findings and the statistical power for comparative analysis. Due to the cohort size, we focused on descriptive and univariate analyses to ensure statistical stability, as multivariate models in small samples can lead to unstable hazard ratios. Furthermore, we were unable to perform a robust statistical analysis on the relationship between time to irAE onset and survival outcomes, which remains a topic of significant interest in the literature [5,16]. Additionally, our results may be subject to guarantee-time bias (or lead-time bias); since patients must survive long enough to develop an irAE to be included, there is a risk of overestimating the survival benefit. However, the magnitude of the 5-year survival rate suggests a biological effect that potentially transcends this bias. Finally, the lack of an internal control group of irAE-negative patients prevents direct hazard ratio comparisons. Nevertheless, this multicenter real-world data provides valuable clinical insights into the long-term survival of patients who must discontinue immunotherapy due to toxicity.

## 5. Conclusions

Patients with metastatic NSCLC who discontinue nivolumab due to irAEs exhibit promising survival that far exceeds historical benchmarks. Our finding of a 27% 5-year OS rate suggests that treatment-limiting irAEs are a powerful surrogate for durable immune responses. Clinicians should recognize that while these events require management, they often herald a favorable long-term prognosis, even after therapy cessation.

## Figures and Tables

**Table 1:** Distribution of Sociodemographic and Clinical Characteristics.

Variables	N	%
Age		
Mean ± SD	60.44 ± 9.87	
Median (min–max)	62.0 (39–81)	
<60	10	40.0
>60	15	60.0
ECOG Performance Status		
0	10	40.0
1	13	52.0
2	2	8.0
Smoking Status		
Former smoker	21	87.5
Current smoker	3	12.5
Histology		
Adenocarcinoma	14	56.0
SCC (Squamous Cell Carcinoma)	8	32.0
Other	3	12.0
Lung Metastasis		
No	8	32.0
Yes	17	68.0
Liver Metastasis		
No	19	76.0
Yes	6	24.0
Bone Metastasis		
No	13	52.0
Yes	12	48.0
CNS Metastasis		
No	19	76.0
Yes	6	24.0
Adrenal Metastasis		
No	22	88.0
Yes	3	12.0
irAEs During Nivolumab Treatment		
Pneumonitis	12	48.0
Colitis	6	24.0
Adrenalitis	1	4.0
Hepatitis	1	4.0
Myositis	1	4.0
Hematological	1	4.0
Dermatological	3	12.0
irAE Grade		
Grade-1	1	4.0
Grade-2	11	44.0
Grade-3	11	44.0
Grade-4	2	8.0
Timing of Metastasis		
De novo metastatic	16	64.0
Recurrent metastatic	9	36.0
Prior Therapies for Stage IV		
0 (None)	4	16.0
1 line	18	72.0
2 lines	3	12.0
PD-L1 Levels		
<1%	11	45.8
1–49%	4	16.7
>49%	4	16.7
Unknown	5	20.8
Response to Nivolumab		
SD (Stable Disease)	5	20.0
PR (Partial Response)	16	64.0
PD (Progressive Disease)	4	16.0
Subsequent Therapy after Nivolumab		
No	13	52.0
Yes	12	48.0
Progression		
No	11	44.0
Yes	14	56.0
Mortality		
Alive	10	40.0
Dead	15	60.0
Follow up (months)		
Mean ± SD	33.08 ± 22.14	
Median (min–max)	33.26 (6.13–116.77)	

**Table 2:** Overall Survival (OS) Analysis.

Variables	2-Year OS (%)	5-Year OS (%)	Median OS (Months) (95% CI)	*p*
Overall	78.9	27.0	35.73 (30.06–41.40)	
Age				
<60	80.0	-	38.16 (31.66–44.65)	0.823
>60	76.6	12.8	34.96 (25.20–44.73)	
ECOG Performance Status				
0	80.0	26.7	57.56 (33.37–81.75)	0.124
1	83.1	-	34.96 (22.55–47.37)	
2	-	-	12.06 (Not Reached)	
Smoking Status				
Former Smoker (Quit)	80.7	17.4	38.16 (25.10–51.22)	0.179
Current Smoker	-	-	24.70 (24.32–25.07)	
Histology				
Adenocarcinoma	69.8	40.7	38.16 (18.46–57.84)	0.694
SCC	87.5	-	38.83 (25.10–42.56)	
Other	100.0	-	48.56 (Not Reached)	
Lung Metastasis				
No	62.5	33.3	35.73 (18.42–53.08)	0.660
Yes	88.2	-	34.96 (28.28–41.65)	
Liver Metastasis				
No	89.5	17.6	38.50 (25.27–51.42)	**0.005**
Yes	44.4	-	23.93 (0.67–47.19)	
Bone Metastasis				
No	83.9	23.0	38.16 (26.15–50.18)	0.344
Yes	74.1	-	33.83 (18.92–48.73)	
CNS Metastasis				
No	84.2	10.5	35.73 (30.43–41.03)	0.620
Yes	66.7	-	33.83 (Not Reached)	
Adrenal Metastasis				
No	80.4	-	38.16 (32.41–43.91)	0.833
Yes	66.7	33.3	29.43 (1.64–57.22)	
irAEs During Nivolumab				
Pneumonitis	91.7	-	48.56 (27.70–69.43)	0.221
Colitis	62.5	-	29.43 (17.95–40.91)	
Others	71.4	35.7	34.96 (11.50–58.42)	
irAE Grade				
Grade-2	91.7	21.8	48.56 (24.36–72.76)	0.137
Grade-3	71.6	-	29.43 (22.01–36.85)	
Grade-4	50.0	-	13.36 (Not Reached)	
Timing of Metastasis				
De novo metastatic	67.5	-	29.43 (20.79–38.07)	**0.012**
Recurrent metastatic	100.0	27.8	57.56 (42.80–72.32)	
Initial Response to Nivolumab				
SD	60.0	-	24.70 (0.00–58.55)	0.198
PR	85.9	28.9	38.50 (34.01–42.99)	
PD	75.0	-	33.83 (1.24–66.41)	
Subsequent Therapy				
Not Received	92.3	20.8	38.50 (37.66–39.33)	0.155
Received	65.6	-	24.70 (16.89–32.50)	

Bold values indicate statistically significant *p*-values (*p* < 0.05).

**Table 3:** Progression-Free Survival (PFS) Analysis.

Variables	2-Year PFS (%)	Median PFS (Months) (95% CI)	*p*
Overall	40.1	16.23 (2.05–30.40)	
Age			
<60	30.0	11.66 (9.91–12.42)	0.827
>60	46.7	16.23 (Not Reached)	
ECOG Performance Status			
0	50.0	10.73 (Not Reached)	**0.001**
1	40.3	23.50 (4.32–42.67)	
2	-	3.23 (Not Reached)	
Smoking Status			
Former Smoker (Quit)	50.4	-	0.212
Current Smoker	-	10.30 (2.24–18.35)	
Histology			
Adenocarcinoma	45.5	16.23 (Not Reached)	0.508
SCC	25.0	9.53 (6.16–12.90)	
Other	66.7	-	
Lung Metastasis			
No	37.5	16.23 (0.00–33.92)	0.848
Yes	44.3	11.16 (8.02–14.30)	
Liver Metastasis			
No	47.8	23.50 (Not Reached)	**0.049**
Yes	16.7	3.23 (0.00–9.55)	
Bone Metastasis			
No	60.6	-	**0.018**
Yes	14.1	7.86 (4.25–11.48)	
CNS Metastasis			
No	48.3	23.50 (Not Reached)	0.409
Yes	16.7	10.73 (9.09–12.37)	
Adrenal Metastasis			
No	40.7	16.23 (0.00–32.55)	0.582
Yes	33.3	9.53 (0.00–19.61)	
irAEs During Nivolumab			
Pneumonitis	54.5	-	0.427
Colitis	16.7	9.53 (0.00–25.13)	
Others	42.9	11.16 (7.65–14.67)	
irAE Grade			
Grade-2	66.7	-	0.190
Grade-3	20.5	10.73 (8.94–12.52)	
Grade-4	-	9.80 (Not Reached)	
Timing of Metastasis			
De novo metastatic	30.5	11.16 (3.78–18.54)	0.531
Recurrent metastatic	55.6	-	
Initial Response to Nivolumab			
SD	-	9.53 (2.97–16.08)	**0.009**
PR	53.0	-	
PD	25.0	3.23 (Not Reached)	
Subsequent Therapy			
Not Received	80.8	-	**<0.001**
Received	-	7.86 (4.41–11.31)	

Bold values indicate statistically significant *p*-values (*p* < 0.05).

**Table 4:** Univariate Cox Regression Analysis for Progression Risk.

Variables	*n* (%)	HR (95% Cl)	*p*
ECOG Status			**0.004**
0	4 (16%)	reference	
1	14 (56%)	0.98 (0.31–3.09)	0.974
2	7 (28%)	22.07 (1.82–266.47)	**0.015**
Bone Metastasis			
No	21 (84%)	reference	**0.026**
Yes	4 (16%)	3.52 (1.16–10.69)	
Subsequent Therapy			
No	15 (60%)	reference	**0.001**
Yes	10 (40%)	30.19 (3.72–244.97)	

Bold values indicate statistically significant *p*-values (*p* < 0.05).

## Data Availability

The data presented in this study are available on request from the corresponding author. The data are not publicly available due to privacy and ethical restrictions regarding patient confidentiality.
